# Comprehensive bioinformatics analyses reveal immune genes responsible for altered immune microenvironment in intervertebral disc degeneration

**DOI:** 10.1007/s00438-022-01912-3

**Published:** 2022-06-29

**Authors:** Bao Hai, Qingpeng Song, Chuanchao Du, Tianli Mao, Fei Jia, Yu Liu, Xiaoyu Pan, Bin Zhu, Xiaoguang Liu

**Affiliations:** 1 Department of Orthopedics, Beijing Friendship Hospital, Capital Medical University, Beijing, 100050 China; 2 Department of Orthopedics, Peking University Third Hospital, No. 49 North Garden Street, Haidian District, Beijing, 100191 China

**Keywords:** Intervertebral disc degeneration, Low back pain, Immune-associated disease, Biomarkers, Weighted gene co-expression network analysis

## Abstract

We sought to identify novel biomarkers and related mechanisms that might shape the immune infiltration in IDD, thereby providing novel perspective for IDD diagnosis and therapies. Gene expression data sets GSE124272 (for initial analysis) and GSE56081 (for validation analysis) involving samples from IDD patients and healthy controls were retrieved from the Gene Expression Omnibus (GEO) database. Immune genes associated with IDD were identified by GSEA; module genes that exhibited coordinated expression patterns and the strongest positive or negative correlation with IDD were identified by WGCNA. The intersection between immune genes and module genes was used for LASSO variable selection, whereby we obtained pivotal genes that were highly representative of IDD. We then correlated (Pearson correlation) the expression of pivotal genes with immune cell proportion inferred by CIBERSORT algorithm, and revealed the potential immune-regulatory roles of pivotal genes on the pathogenesis of IDD. We discovered several immune-associated pathways in which IDD-associated immune genes were highly clustered, and identified two gene modules that might promote or inhibit the pathogenesis of IDD. These candidate genes were further narrowed down to 8 pivotal genes, namely, MSH2, LY96, ADAM8, HEBP2, ANXA3, RAB24, ZBTB16 and PIK3CD, among which ANXA3, MSH2, ZBTB16, LY96, PIK3CD, ZBTB16, and ADAM8 were revealed to be correlated with the proportion of CD8 T cells and resting memory CD4 T cells. This work identified 8 pivotal genes that might be involved in the pathogenesis of IDD through triggering various immune-associated pathways and altering the composition of immune and myeloid cells in IDD patients, which provides novel perspectives on IDD diagnosis and treatment.

**Supplementary Information:**

The online version contains supplementary material available at 10.1007/s00438-022-01912-3.

## Introduction

Low back pain (LBP) is ranked among the top-3 causative factors responsible for disability in developed countries, whose victims are still growing globally (Murray et al. 2012). Nearly 8 in 10 adults will experience such disease throughout their life span (Cannata et al. 2020), and most of them have to struggle with LBP associated disadvantages, such as reduced life quality, movement disability and potential unemployment (Andersson 1999). Thus, LBP has imposed an excessive burden on the economy and the society. Currently, the therapeutic outcomes of LBP are impeded by limited understanding of the underlying mechanisms (Vergroesen et al. 2015). Nevertheless, this refectory symptom has been proposed to be strongly associated with intervertebral disc degeneration (IDD) (de Schepper et al. 2010), among other factors, such as genetic background or environmental impact (Cannata et al. 2020).

IDD is a long-term pathological process that could be partially characterized by a gradual loss of proteoglycans and liquid content within the intervertebral disc (IVD) (Cannata et al. 2020). Although the pathophysiology of IDD has been established, the underlying etiology has remained obscur; thus, it is imperative to gain a deeper insight into the way by which IDD is initiated and developed. Normally, nucleus pulposus (NP) is isolated from the immune system by the surrounding intact IVD structure (for example, annulus fibrosus, AF); upon impairment, NP is exposed to the immune system, leading to a series of auto-immune responses which play a fundamental role in the progression of IDD (Sun et al. 2020). For decades, extensive efforts have been put into clarifying the association between IDD and immune cells (Risbud and Shapiro 2014). For instance, T cells, B cells and neutrophils might be implicated in the NP-exposure-triggered auto-immune response (Wang and Samartzis 2014). In contrast, the activity of natural killer (NK) cells was found to be significantly lower in patients with lumbar disc herniation than that in healthy volunteers, indicating that the patients might experience stress resulted from pain or other discomforts (Sato et al. 2002). The landscape of immune infiltration and diagnostic markers for IDD have been revealed recently (Wang et al. 2021). Although macrophage was deemed the most important player in IDD according to numerous studies involving human participants (Silva et al. 2019; Wang et al. 2021), the implication of other immune cells in the development of IDD is still ill-defined, especially in the clinical setting. Hence, details regarding altered IDD immune microenvironment are worth discussing and deserve further investigation.

Given the foregoing, the present study aimed at exploring altered immune pathways-based biomarkers that are involved in the pathogenesis of IDD, and their association with immune cell infiltration. Bioinformatics is an emerging interdisciplinary field that borrows strengths from both biological knowledge and computational potential, and has been extensively utilized to unravel biologically significant markers that are hidden within the tremendous biological data (Cai et al. 2020; Yan et al. 2021). The advent of gene chip or microarray allowed highly efficient acquisition of biological data, and GEO (Gene Expression Omnibus) database serves as a global repository of the high-throughput information generated by gene chip or micro array, and so forth, allows in-depth analysis of the differences between IDD patients and healthy volunteers. In the current comprehensive analysis, several bioinformatics algorithms were incorporated: GSEA was used to interpret the biological significance between different phenotypes in a gene-expression-dependent manner (Subramanian et al. 2005), and, most importantly, unearth immune-associated genes responsible for IDD. WGCNA has long been used to detect gene modules (wherein genes exhibit coordinated expression patterns), and subsequently associate biologically significant modules with clinical traits; such an algorithm tends to find hub genes that play central roles in a regulatory network (Langfelder and Horvath 2008). CIBERSORT is a v-support vector regression-based algorithm used to infer immune cell composition in bulk RNA-seq samples by referring to an immune-cell signature matrix of 22 human hematopoietic cell phenotypes (Newman et al. 2015), which enabled us to associate pivotal IDD markers with altered IDD immune environment. Through combined use of the-above-mentioned bioinformatics methods, we expect to find out immune signature genes that are involved in the altered immune cell composition and in the pathogenesis of IDD. Through combined utilization of various bioinformatics and machine learning tools, we hope to provide novel perspectives regarding IDD diagnosis and treatment in the context of immune infiltration.

## Methods

### Data collection

Gene expression data sets used in the current study were obtained from the Gene Expression Omnibus database (GEO; http://www.ncbi.nlm.nih.gov/geo/) on January 5th, 2021. Two GEO series were chosen for incorporation into this study, including an initial analysis set (GSE124272) (Wang et al. 2019) and a validation set (GSE56081) (Liu et al. 2015). GSE124272 contained whole blood samples collected from 8 IDD patients (IDD) and equal number of healthy volunteers (HV) was used in the analyses (including GSEA, WGCNA, GO/KEGG enrichment analysis, LASSO feature selection). GSE56081 included nucleus pulposus tissues derived from 5 IDD patients and 5 controls and was used for validating the biological significance of the pivotal IDD biomarkers identified in GSE124272. Moreover, CIBERSORT inference of the composition of immune cells was executed in both the initial and validation data sets, whereby comparisons of the immune-regulatory roles of pivotal IDD biomarkers could be made across IDD samples of different origins. The overall design of this study is shown as a flow chart in Supplementary Fig. S1.

### Gene set enrichment analysis (GSEA)

To reveal the alerted biological functions contributing to the pathogenesis of IDD in a gene-expression dependent fashion, we first conducted a GSEA using clusterProfiler (3.16.1) (Yu et al. 2012) in R (version 4.0.2). In brief, genes were ranked based on their differences in expression between IDD and HV; in this manner, gene expression could be associated with phenotypes (IDD or HV). Next, the ranked gene list was fed to GSEA algorithm, and queried against GEO data base to associate gene expression with functional enrichment. In particular, genes were examined throughout the ranked gene list, during which the running sum statistic was raised as long as a gene in the queried pathway was encountered; otherwise, the statistics was reduced by the algorithm, and an Enrichment score (ES) representing the maximum deviation of running-sum statistic from zero could be calculated. Leading-edge subsets that contributed greatly to the *ES* of immune-associated pathways were extracted, and the intersection among them was defined as immune genes.

### Weighted correlation network analysis (WGCNA)

Co-expression network was constructed through WGCNA (1.69) (Langfelder and Horvath 2008) in R. We first filtered non-varying genes which could be generally considered as noise (based on a 75% median absolute deviation threshold), whereby the robustness and effectiveness of WGCNA algorithm was improved. To construct a scale-free network, the gene co-expression similarity S_kj_ between two arbitrary genes (nodes) m and n were calculated using *S*_*kj*_ =|cor(*k*, *j*)|. The pickSoftThreshold function in WGCNA package was used to screen ideal soft thresholding power *β* by calculating the degree of independence as β gradually increases, an optimized *β* was picked when the degree of independence exceeded a 0.85 threshold, after which S_kj_ was raised by *β*, whereby the co-expression similarity was transformed into and adjacency: *a*_*kj*_ =|*S*_*kj*_|^*β*^, resulting in the construction of a topological overlap matrix. Next, module detection was carried out with a minimal module size being set as 30, dynamic tree cut method was subsequently applied to merge highly similar modules. The finalized modules were then related to external phenotypes (IDD or HV).

### Pathway enrichment analysis (GO/KEGG)

Modules with the highest positive/negative correlation with IDD were named pivotal modules and subjected to pathway enrichment analysis, which was performed using clusterProfiler, whereby biological significance of two pivotal modules were interpreted. Next, we defined the union of genes within pivotal modules as module genes. The intersection between modules genes (identified by WGCNA) and immune genes (revealed by GSEA) were named immune signature genes and underwent further investigation in our subsequent analyses.

### LASSO feature selection

To ensure the robustness of subsequent analyses, LASSO (least absolute shrinkage and selection operator) algorithm was performed on immune signature genes to screen candidates for machine learning modeling. LASSO was performed using glmnet package (Friedman et al. 2010) (version 4.0.2) in R. The features selected by LASSO bears a greater biological significance and were named pivotal genes before being used in machine learning based model validation.

### Machine learning-based model validation

For further confirmation of the biological significance concerning pivotal genes, 5 machine learning predictive models were applied to both GSE124272 and GSE56081 data sets, with pivotal genes being used as features. The tenfold cross validation method was used to evaluate the combinations of hyperparameters. The main parameters of the 5 models were as follows: (i) the *λ* penalty coefficient of LASSO was set to 0.2; (ii) the radial basis function (RBF) was set to the kernel function in SVM; (iii) the number of sub-decision trees was set to 500 in Random forest (RF); (iv) the eXtreme Gradient Boosting (Xgboost) had the maximum depth of 10, and the learning rate was 0.001; (v) The back propagation neural network (BPNN) model had one single hidden layer, and the number of neurons was set to 10. The activation function of hidden layer and output layer were set to ReLU and Sigmoid function, respectively. The cross-entropy function was set to loss function. Finally, the model was trained through batch gradient descent method with 1000 iterations, and the learning rate was 0.001. Receiver operating characteristic curve (ROC) and area under curve (AUC) were used to evaluate the performance of model. Machine-learning model validation was performed in python (version 3.7.6).

### Correlation analysis of pivotal gene expression and IDD immune environment

To better understand the altered immune environment in the pathogenesis of IDD, CIBERSORT algorithm (Newman et al. 2015) (cell-type Identification by Estimating Relative Subsets of Known RNA Transcripts) was used to infer immune cell composition in both training and validation data sets, and then we measured the Pearson-correlation coefficient between the level of immune infiltration (proportion of a type of immune cell) and the expression of 8 pivotal genes across all IDD patients enrolled in this study. All gene-cell pairs with significant correlation coefficient were subsequently visualized using Cytoscape (Shannon et al. 2003) (version 3.6.1), and comparisons were made between training set and validation set.

## Results

### GSEA-based identification of immune genes responsible for IDD

**Fig. 1:**
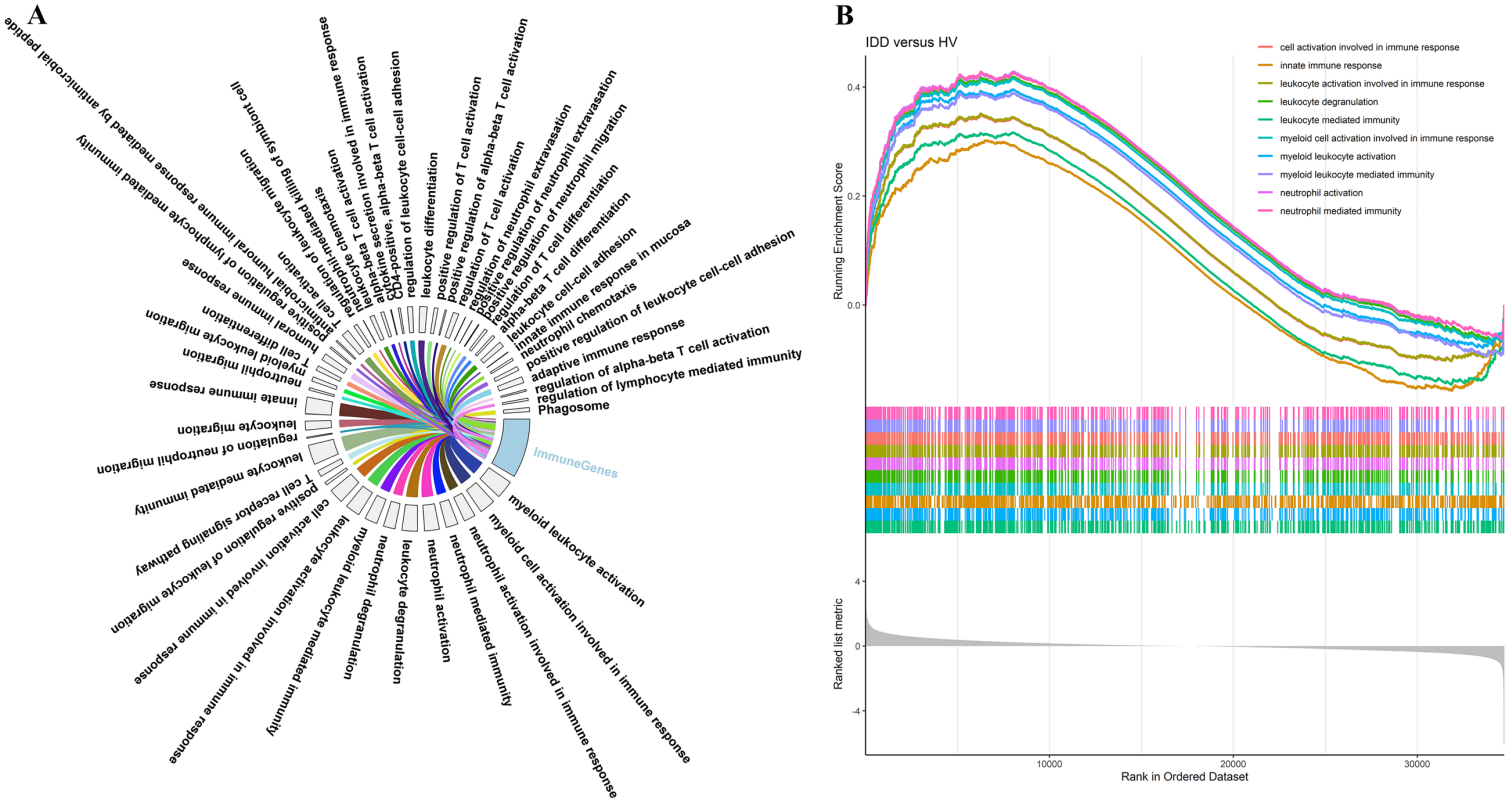
Identification of immune genes involved in IDD. **A** Involvement of immune genes in GSEA-immune-associated pathways were shown in circos plot, the spill-over from “Immune Genes” sector to other sectors indicates the distribution of immune genes in different immune-associated pathways, the area of a sector corresponds to the number of genes in that category. **B** Enrichment plot of the top-10 representative immune-associated GSEA pathways, the running-sum statistic of each pathway was denoted by different colors, the *ES* for each pathway was above zero

To identify the immune-related genes that are potentially involved in IDD, we performed GSEA analysis based on GO and KEGG source databases. The GSEA–GO and GSEA–KEGG results were provided in Supplementary Tables S1 and S2. We extracted immune-associated pathways from GSEA results, and defined the intersection of leading-edge gene sets of all immune-associated pathways as immune genes. The involvement of immune genes in GSEA-immune-associated pathways were shown in circos plot (Fig. 1A); the top-10 representative immune-associated pathways (with a predominant number of immune genes) were “cell activation involved in immune response”, “innate immune response”, “leukocyte activation involved in immune response”, “leukocyte degranulation”, “leukocyte mediated immunity”, “myeloid cell activation involved in immune response”, “myeloid leukocyte activation”, “myeloid leukocyte mediated immunity”, “neutrophil activation” and “neutrophil mediated immunity”. Enrichment plot of the above-mentioned pathways is shown in Fig. 1B; it is notable that all of these pathways achieved positive enrichment score (ES) in the IDD *versus* HV comparison, suggesting that the genes involved in these predominant immune-associated pathways were up-regulated in IDD patients, showing the biological significance of immune genes retrieved from these pathways.

### Detection of IDD-associated co-expression modules by WGCNA

**Fig. 2:**
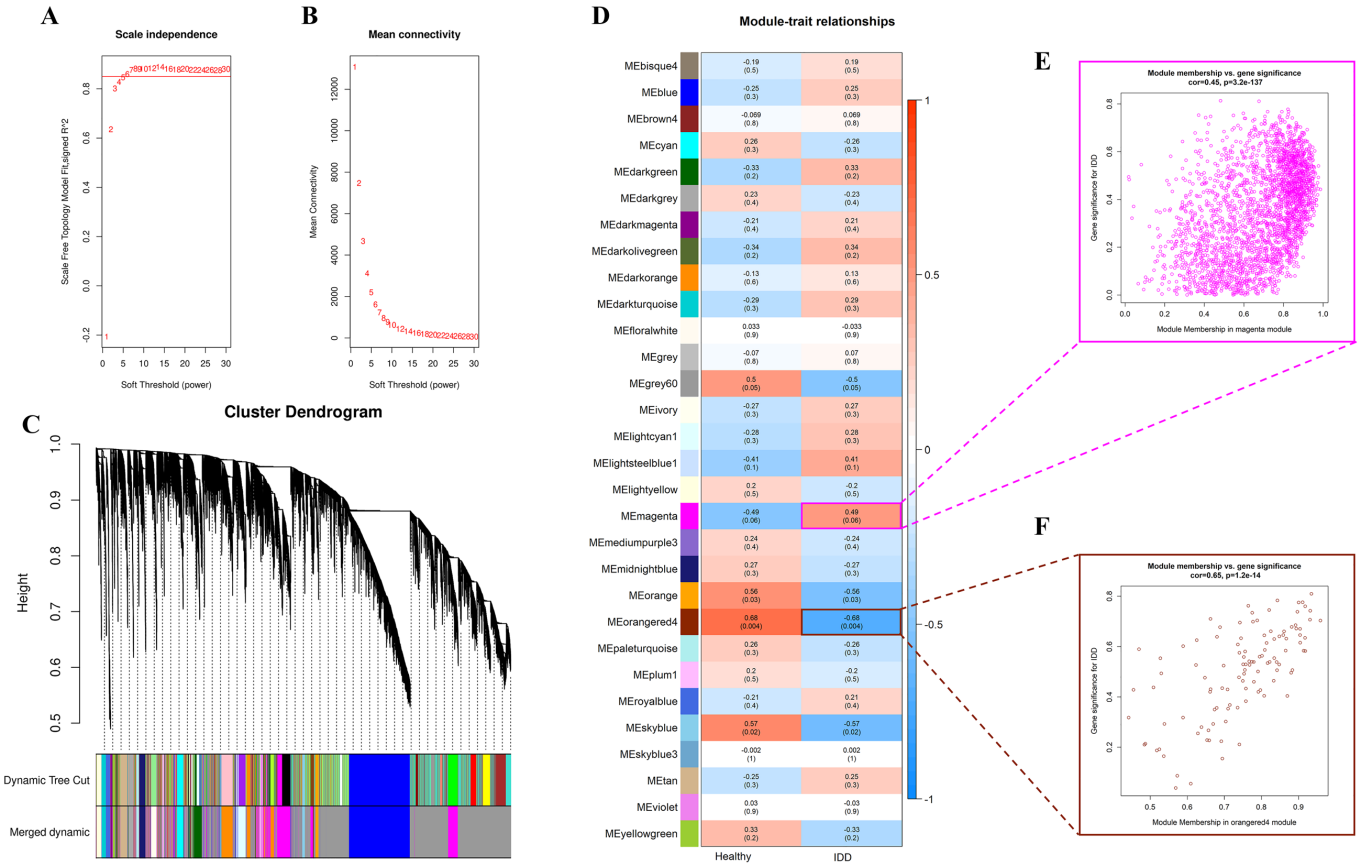
Identification of IDD-associated gene modules. **A** Increase in soft threshold power was accompanied by elevated scale-free fit index; 6 was the lowest power β that corresponded to a scale-free fit index ≥ 0.85 (the threshold was indicated by a red horizontal line). **B** Mean connectivity was gradually reduced with the increased power, the choice of the power (*β* = 6) retained considerable mean connectivity. **C** Dendrogram and barcode plot showed that the genes were classified into different gene modules. Modules with high similarity were further merged into larger modules. Different colors in barcode plot corresponded to different modules. **D** Heatmap of module-phenotype relationships reported the correlation and significance (*p* value in parenthesis) between modules and the phenotypes of the samples. The red or blue cube represented a positive or negative correlation. The magenta or orangered4 module had the strongest positive or negative correlation with the IDD phenotype. **E, F** Scatter plots (gene significance versus module membership) of magenta and orangered4 modules

**Fig. 3:**
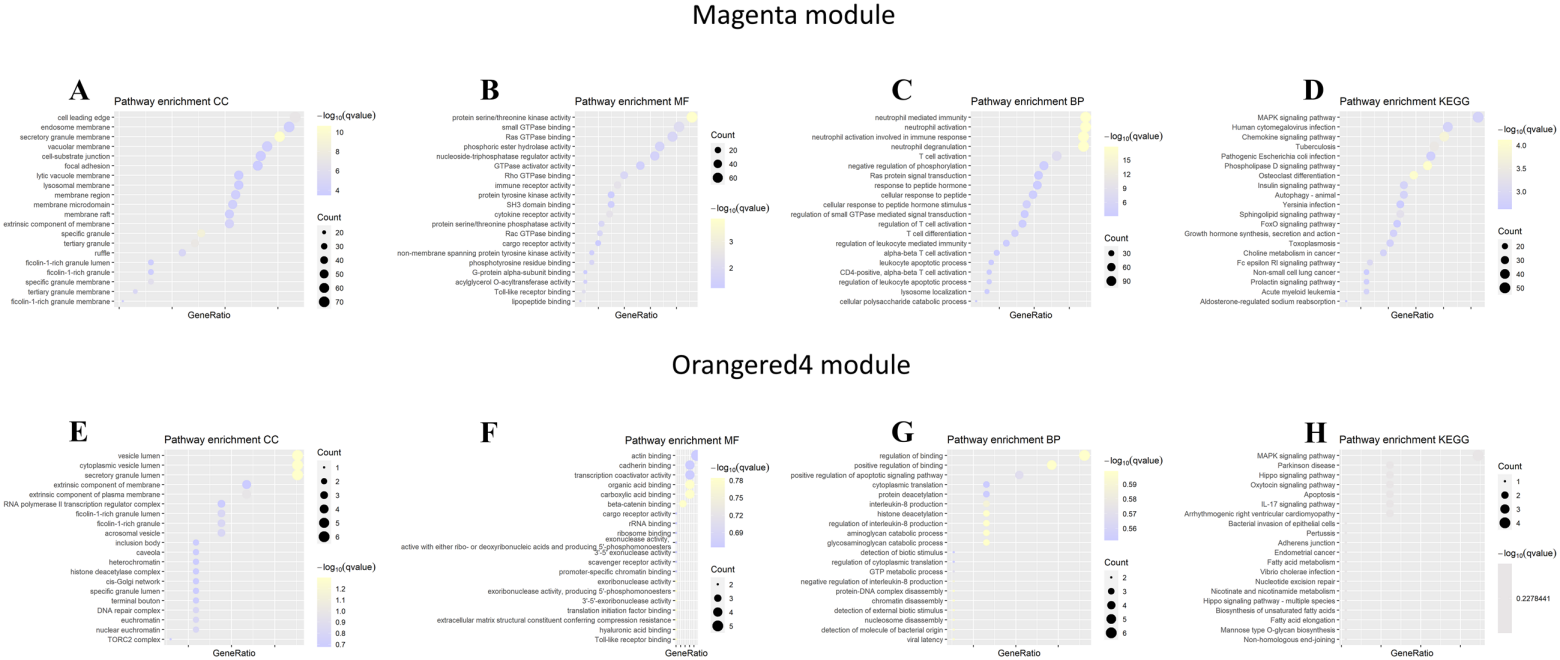
Functional enrichment analysis of magenta and orangered4 module genes. Module genes in magenta and orangered4 modules were queried against GO or KEGG database, enrichment categories of both modules are shown in (**A**, **E**) cellular component; **B**, **F** molecular function; **C**, **G** biological process and **D**, **H** KEGG pathway, respectively. The dot size corresponded to the count of differentially expressed genes, and enrichment significance was displayed by a color gradient

To construct a scale-free network, an optimal power was first screened; as depicted in Fig. 2A, the soft-thresholding power was determined to be 6 by the algorithm when scale-free fit index first reached a 0.85 threshold, where the mean connectivity remained relatively high. Afterwards, a topological overlap matrix was generated; a series of modules were subsequently detected by hierarchical clustering, and we narrowed down the number of modules using Dynamic Tree Cut algorithm, whereby modules with high similarity were merged (Fig. 2C). Next, the merged modules were correlated to sample clinical traits (IDD or HV), the resulting correlation and *p* value (in parenthesis) were displayed in a heatmap (Fig. 2D), among which magenta module and orangered4 module displayed the strongest positive or negative correlation with IDD phenotype and were chosen for further investigation. The scatter plots of gene significance for IDD *versus* module membership in magenta or orangered4 modules are shown in Fig. 2E, F, respectively. The biological significance of these two modules was interpreted using GO or KEGG pathway enrichment analysis. As shown in Fig. 3A–D, Magenta module genes were most significantly enriched in “secretory granule membrane” (GO-Cellular component, CC); “protein serine/threonine kinase activity (GO-Molecular function, MF)”; “neutrophil mediated immunity”, “neutrophil activation”, “neutrophil activation involved in immune response”, “neutrophil degranulation” (GO-Biological process, BP); “Phospholipase D signaling pathway”, and “Osteoclast differentiation” (KEGG). As shown in Fig. 3E–H, Orangered4 module genes were most significantly enriched in “vesicle lumen”, “cytoplasmic vesicle lumen”, “secretory granule lumen” for CC; “organic acid binding”, “carboxylic acid binding”, “beta-catenin binding” for MF; “regulation of binding”, “positive regulation of binding”, “histone deacetylation”; “regulation of interleukin-8 production”, “aminoglycan catabolic process”, and “glycosaminoglycan catabolic process” for BP, while the significance of enrichment of Orangered4 module genes under each category of KEGG was identical. Nevertheless, “MAPK signaling pathway” had the highest GeneRatio (Number of genes involved in a given pathway/Total number of queried genes), and could represent the KEGG enrichment of Orangered4 module genes. We then defined the module genes as the intersection of genes in these two modules for subsequent analyses.

### Selection for pivotal immune-associated players in the pathogenesis of IDD

**Fig. 4:**
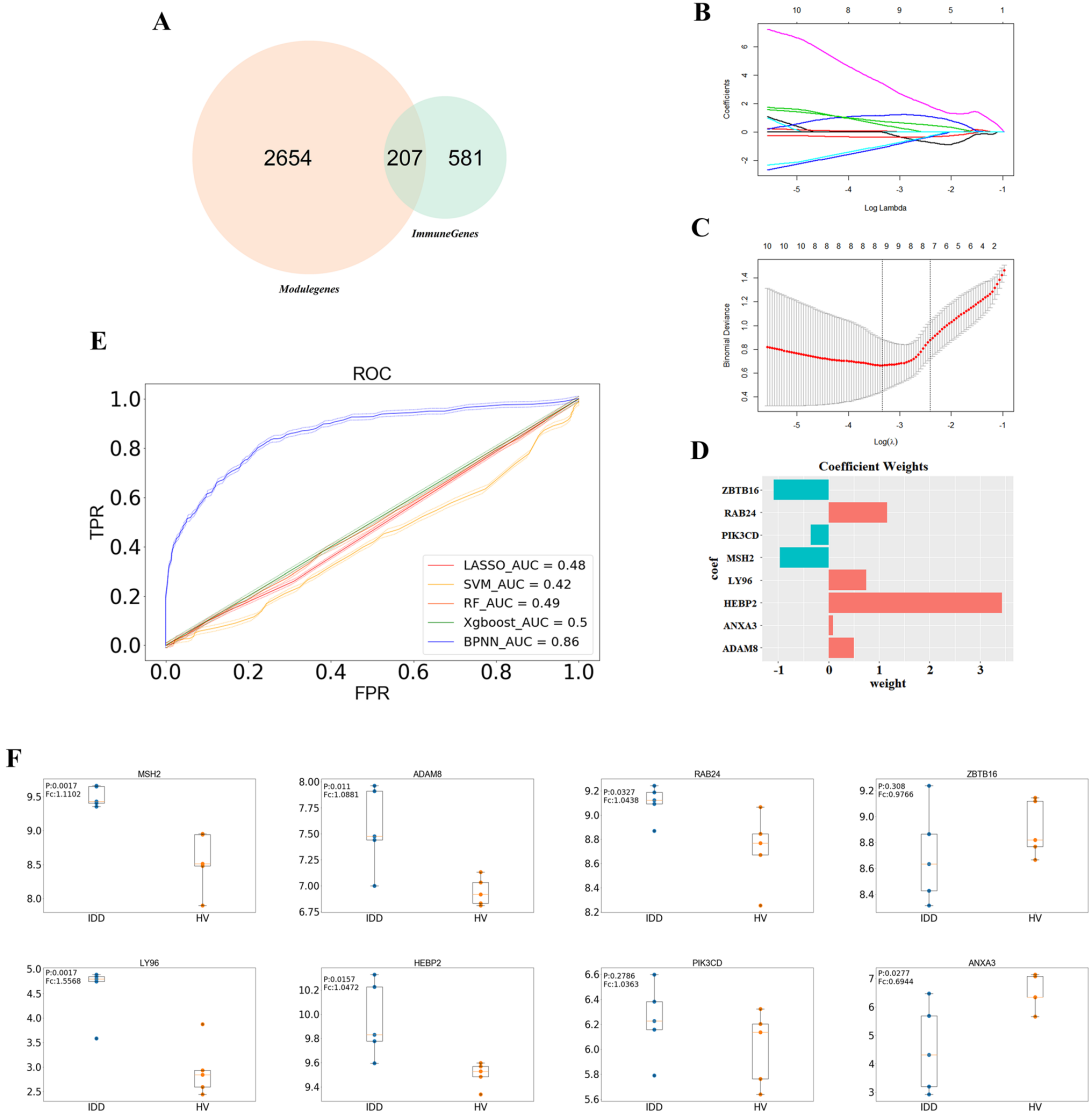
Selection of pivotal genes and machine learning model validation. **A** Intersection between module genes (2861 genes, identified by WGCNA) and immune genes (788 genes, identified by GSEA) were named immune signature genes (207 genes). **B**, **C** LASSO penalized model demonstrated that when Log *λ* =− 3.339, the minimum binomial deviance was obtained. **D** Coefficient of 8 selected pivotal genes (with non-zero coefficients when Log *λ* = − 3.339). **E** ROC curve demonstrated the prediction effect of 5 machine learning validation models on the validation set, the 95% confidence interval was marked with dotted lines. **F** Expression patterns of 8 pivotal genes in validation set (tissue samples). *P* *p* value, *Fc* log-fold change (IDD versus HV)

Based on the results of GSEA (leading edge genes in immune-associated pathways responsible for the pathogenesis of IDD), and WGCNA (IDD-associated genes with coordinated expression patterns), we defined the intersection of immune genes and module genes as immune signature genes (Fig. 4A), which contained 207 genes. To ensure the robustness of the current study, we used LASSO algorithm to screen for pivotal genes with higher biological significance from 207 immune signature genes. The LASSO path plot is shown in Fig. 4B, with the increase of λ parameter, the coefficients were shrunk towards zero, and λ corresponding to the minimum binomial deviance was chosen as the penalty coefficient (Fig. 4C), the remaining 8 pivotal genes (with non-zero coefficients estimates) were named pivotal genes, and their coefficient weights are shown in Fig. 4D. The expression of 8 pivotal genes in validation set was shown in boxplot (Fig. 4F); aside from ANXA3 and ZBTB16 which were down-regulated in IDD patients, other pivotal genes were significantly up-regulated in IDD patients, especially MSH2 and LY96 (with P value less than 0.01). To investigate whether the 8 pivotal genes could constitute a diagnostic model for distinguishing IDD patients from HV controls, five machine learning algorithms, namely, “LASSO”, “SVM”, “RF”, “Xgboost” and “BPNN” were run. As shown in Fig. 4E, the area under curve (AUC) obtained with the five machine learning models ranged between 0.48 and 0.86 and the highest AUC of 0.86 was obtained with the BPNN model. These results indicated that the 8 pivotal genes could be used as a diagnostic model for distinguishing between IDD patients and healthy individuals.

### Correlation between pivotal genes and immune cell composition

**Fig. 5:**
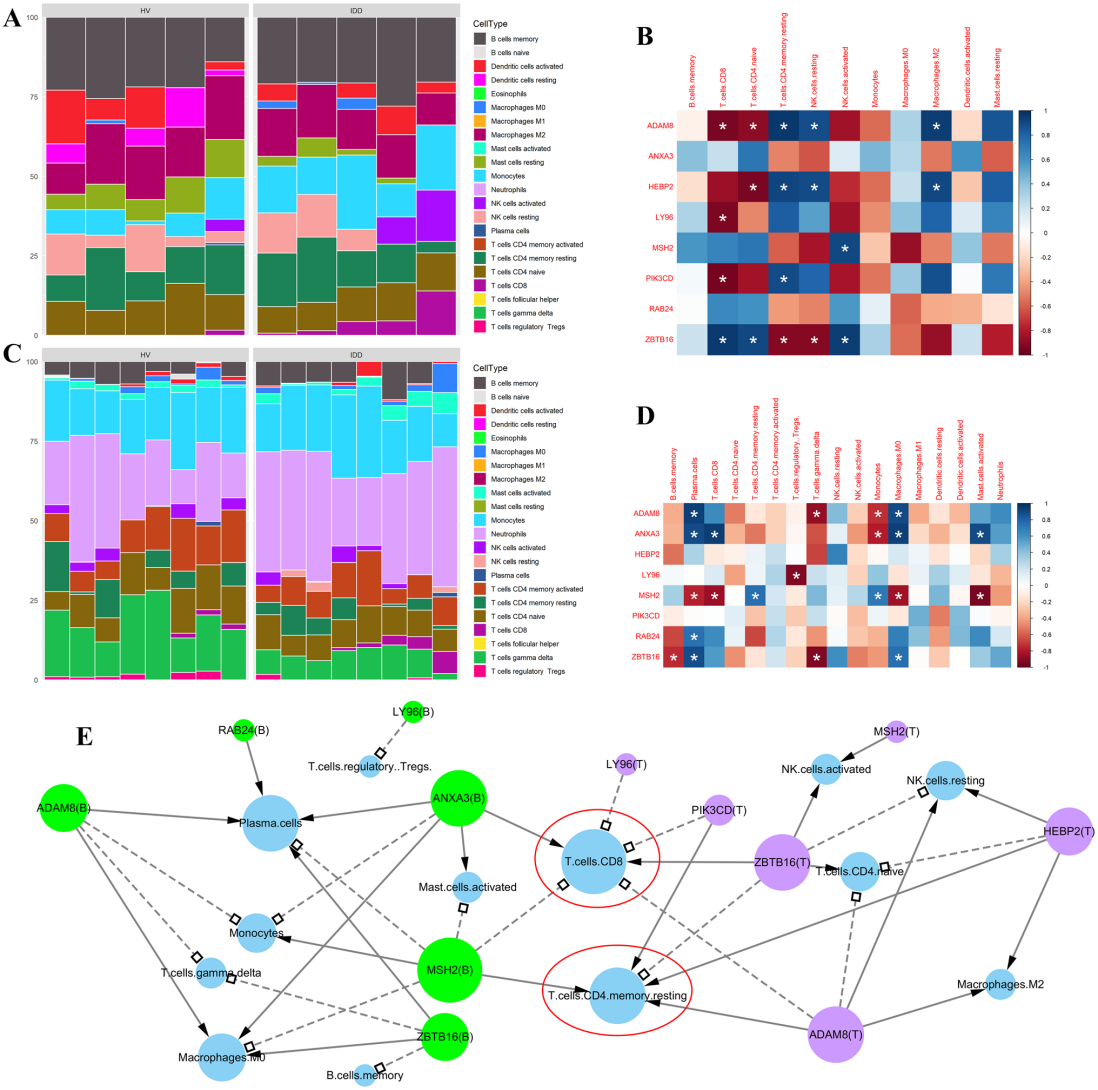
Correlation between the immune cell composition and the expression of pivotal genes. Stacked barplots demonstrate the inferred immune cell composition of IDD and HV groups in **A** validation set (tissue samples) and **C** training set (blood samples). The heatmaps show the correlation between pivotal genes (rows) and immune cells (columns) in **B** validation set and **D** training set, where positive and negative correlations were denoted by blue or red blocks, respectively. Significant pairwise correlations (with *p* value less than 0.05) were highlighted by asterisks. **E** All gene-cell pairs corresponding to significant correlations were summarized in an interaction network, where significant positive or negative correlations were represented by arrows with solid lines or squared arrows with dash lines, respectively. The green, purple or blue nodes correspond to pivotal genes expression in training set/pivotal genes expression in validation set/proportion of immune cells. Characters in parentheses indicate the expression of pivotal genes in “B” (blood samples, training set) or “T” (tissue samples, validation set). Nodes with larger size possess an increased number of edges. Nodes highlighted by red circles represented the proportion of CD8 T cells and resting memory CD4 T cells, which was associated with pivotal genes in both blood and tissue samples.

Immune genes undoubtedly affect the immune processes, which can be reflected by altered immune cell composition. Therefore, we constructed a correlation matrix to visualize the association between the expression of pivotal genes and the proportion of immune cells across different samples based on the two data sets. As shown in Fig. 5A, C), the stacked bar plot represented the accumulated proportion of 22 immune genes in IDD samples inferred by CIBERSORT, and the heatmaps (Fig. 5B, D) showed the correlation between pivotal genes and immune cells in IDD samples, where positive and negative correlations were denoted by blue or red blocks, respectively. The correlations are summarized in Fig. 5E, which demonstrated that the above pivotal genes jointly regulate CD8 T cells and resting memory CD4 T cells across both data sets.

## Discussion

In the present study, we first focused on the GSE124272 containing blood samples; numerous immune-associated pathways that might be responsible for the pathogenesis of IDD were initially identified by GSEA, whereas gene modules that displayed strong correlation with IDD phenotype were detected by WGCNA. Afterwards, 207 immune signature genes were defined on the basis of GSEA and WGCNA, which were subsequently narrowed down to 8 pivotal genes by LASSO feature selection. We then turned our attention to the validation data (GSE56081), and performed cross-validation of 8 pivotal genes using 5 machine learning models, whereby “BPNN” model indicated that the eight gene could be used for distinguishing IDD patients from HV with an AUC of 0.86, which further verified the biological significance of pivotal genes.

Our GSEA results showed that the most representative immune-associated pathways were related to immune response, leukocyte, myeloid and neutrophil, and their positive *ES* in IDD *versus* HV comparison indicated that the above pathways might be activated in the pathogenesis of IDD, along with up-regulation of immune genes involved in these regulatory processes. Specifically, we found that the immune genes were highly clustered in the following pathways: “cell activation involved in immune response”, “innate immune response”, “leukocyte activation involved in immune response”, “leukocyte degranulation”, “leukocyte mediated immunity”, “myeloid cell activation involved in immune response”, “myeloid leukocyte activation”, “myeloid leukocyte mediated immunity”, “neutrophil activation” and “neutrophil mediated immunity”. Aside from macrophage, a mononuclear leukocyte that participates in innate immune response (Danielsson and Eriksson 2021) and IDD (Silva et al. 2019), previous studies demonstrated the activation of leukocytes during NP-associated intervertebral disc impairments (Wang et al. 2021). For instance, in animal IDD model, T-helper cells, T-killer cells and B cells were activated and attracted by NP exudate (Geiss et al. 2007); T cells and neutrophils were proposed to secret molecules that promote inflammation, autophagy or apoptosis (including well-studied TNF and IL-1β), thereby contributing to the IDD (Risbud and Shapiro 2014). Collectively, myeloid leukocyte (neutrophils), leukocytes (macrophages, T cells and B cells) were proposed to be activated and involved in the pathogenesis of IDD; therefore, the GSEA results (especially *ES* that indicated positive correlation between these immune pathways and IDD phenotype) obtained in the present study agreed with previous reports.

The subsequent WGCNA revealed two modules that closely associated with IDD, namely, magenta module that was positively associated with IDD, and orangered4 module that was negatively associated with IDD. Function enrichment analyses showed that, in the category of CC, magenta or orangered4 module genes were most significantly enriched in “secretory granule membrane” or “vesicle lumen”, “cytoplasmic vesicle lumen”, and “secretory granule lumen”. The importance of extracellular vesicles (EV, a heterogeneous mixture of vesicles) in the field of inter-cell communication has been established in recent years (McConnell 2018), especially those that are responsible for immune processes in response to infectious diseases (Hosseini-Beheshti and Grau 2018). Specifically, the release of EV targets several immune cells including T-cells and macrophages in the presence of exogenous pathogen (McConnell 2018). Likewise, secretory granule could serve as a “shuttle” by carrying various host defense peptides, continuous replenishment and release of these “shuttles” are crucial for maintaining the innate host defense (Yokoi et al. 2019). However, the immune regulatory roles of EV and secretory granule in auto-immune responses are still unclear. Considering that the genes in both magenta and orangered4 modules were significantly enriched in “secretory granule membrane”, we hypothesized that secretory granule might be a double-edged sword in the pathogenesis of IDD, while vesicles-associated pathway might be protectives factors to IDD.

With regard to MF terms, magenta or orangered4 module genes were predominantly involved in “protein serine/threonine kinase activity”, “organic acid binding”, “carboxylic acid binding”, and “beta-catenin binding”. Protein kinase B is a well-established serine/threonine kinase whose activation depend greatly on its phosphorylation during the immune activation of T cells (Fabre et al. 2005; Finlay and Cantrell 2011); these might explain the involvement of magenta module genes in “protein serine/threonine kinase activity”, since activated leukocytes might be accompanied by elevated protein serine/threonine kinase activity in IDD patients. The top enriched MF terms for orangered4 module suggested that the ability of interacting selectively and non-covalently with an organic acid or carboxylic acid or beta-catenin might be hampered in IDD patients, among which beta-catenin was proposed as a mediator of immune evasion which are exploited by cancer cells to hide from host immune responses (Du et al. 2020; Sorci et al. 2013). Moreover, activated Wnt/beta-catenin pathway prevents T cells from infiltrating into metastatic melanomas, resulting in local immune exclusion (Pai et al. 2017); therefore, we speculated that beta-catenin might exert protective roles in IDD by calming the inflammation provoked by T cells.

As for the BP category, magenta or orangered4 module genes were mainly clustered in “neutrophil mediated immunity”, “neutrophil activation”, “neutrophil activation involved in immune response”, “neutrophil degranulation”, “regulation of binding”, “positive regulation of binding”, “histone deacetylation”, “regulation of interleukin-8 production”, “aminoglycan catabolic process”, and “glycosaminoglycan catabolic process”. The results of magenta module were highly consistent with that of GSEA, further confirming the roles of these immune cells in the pathogenesis of IDD. In contrast, orangered4 module genes were implicated in BP terms that promotes binding (the selective interaction between molecules). Although not reported, these biological processes may have a profound impact on IDD. Histone deacetylation is a pleiotropic immune regulator that not only participates in the development and differentiation of myeloid, but also regulates the functions of the mature leukocytes (such as macrophage and dendritic cells) through controlling the generation of inflammatory factors (Shakespear et al. 2011), while interleukin-8 is a pro-inflammatory chemokine generated by macrophages (Hedges et al. 2000); thus, the enrichment results showed that orangered4 module genes might counteract the IDD through regulating immune processes induced by histone deacetylation and interleukin-8.

The KEGG enrichment results showed that magenta module genes were mainly involved in “Phospholipase D signaling pathway” and “Osteoclast differentiation”. In most cases, the intracellular activity of phospholipase D remains low, unless the cells were stimulated by cellular stress (Bruntz et al. 2014; Shin et al. 2001), and we presumed that phospholipase D and associated signaling pathway might be activated in response to various cellular stress in IDD, such as mechanical stress (Chooi et al. 2017) and oxidative stress (Hou et al. 2014). Osteoclast is a crucial player in remodeling, maintaining, and repairing of bone tissue (Jayakumar and Di Silvio 2010), and was found activated in IVD with Modic changes (normally occur near the degenerated IVD), which could also be partially attributed to mechanical stress (Rahme and Moussa 2008; Torkki et al. 2016). The enrichment of magenta module genes in these pathways were concordant with their positive correlation with IDD. The significance of enrichment of Orangered4 module genes under each KEGG pathway was identical, among which “MAPK signaling pathway” achieved the highest GeneRatio; such pathway is essential for initiating the innate immunity through participating in cytokine generation, it also plays a fundamental role in lymphocyte differentiation (Krzyzowska et al. 2010). Therefore, we hypothesized that MAPK associated processes might contribute to the anti-IDD effects in orangered4 module.

The combined utilization of GSEA and WGCNA identified several biological significant gene sets, which were considered responsible for the pathogenesis of IDD, and used for selecting pivotal markers (highly representative of IDD). After the selection by LASSO algorithm, we obtained 8 pivotal IDD markers (ANXA3 and ZBTB16 (down-regulated in IDD patients), MSH2 and LY96 (up-regulated in IDD patients with p value less than 0.01), along with ADAM8, HEBP2, RAB24, PIK3CD (up-regulated in IDD patients)) and evaluated their expression in validation set. Among these genes, ADAM8 (A disintegrin and metalloproteinase) was found in IDD tissues (including both NP and AF tissues), and known to be responsible for FN cleavage (FN: fibronectin fragments which increase with the extent of disc degeneration and involved in the initiation of IDD progression) (Oegema et al. 2000; Ruel et al. 2014). Human mesenchymal stem cells induced regeneration of NP cells has emerged as a novel strategy to attenuate the negative effects of NP cell degeneration, and ANXA3 (Annexin A3) was recommended as a maker that determine the success of such regenerative process (Ehlicke et al. 2017). Other pivotal genes, although not reported, were associated with immune responses. ZBTB16 (transcription factor promyelocytic leukemia zinc finger) is indispensable for timely and intense immune response of almost all Natural Killer T cells (Zhang et al. 2015). MSH2 (mutS homolog 2) was originally recognized as a regulator in DNA mismatch repair pathway and its strong correlation with elevated PD-L1 expression and immune infiltration was only revealed recently in lung adenocarcinoma (Jia et al. 2020). LY96 (Lymphocyte antigen 96), also known as MD-2 (Myeloid Differentiation factor 2), is a molecular chaperone of TLR4 (Toll-like receptor 4); the TLR4/MD-2 complex was found to control the early immune responses against bacterial infection (Robison et al. 2019). PIK3CD is a well-studied immune gene that not only shape the development and function of B cells (Wray-Dutra et al. 2018), but also is responsible for the susceptibility of T cells to virus infection (Rodriguez et al. 2019). HEBP2 (Heme Binding Protein 2) and RAB24 (Ras-Related Protein Rab-24) were related to pathways associated with innate immune system according to the gene card (www.genecards.org) database (Stelzer et al. 2016). Collectively, pivotal genes identified in our current study bear the potential to distinguish inflammatory IDD, their biological significance was also validated by the BPNN machine learning models with an AUC of 0.86.

Immune cell infiltration is accountable for the initiation and progression of IDD (Shamji et al. 2010). Considering that our currently identified pivotal genes were highly representative of IDD and strongly associated with immune response, we sought to find their down-stream target immune cells that exert the major immune-pathological effects in IDD. Therefore, we evaluated the correlation between composition of immune cells and the expression of pivotal genes in IDD patients from both data sets. We found that, in blood samples, plasma cells and M0 macrophages were positively correlated with the majority of pivotal genes (including ADAM8, ZBTB16, ANXA3 and RAB24), whereas in tissue sample, pivotal genes (including ZBTB16, MSH2, HEBP2 and ADAM8) were mainly positively correlated with activated NK cells and M2 macrophages. The discrepancies regarding the correlation between pivotal genes and immune cells components across different origin of IDD samples (blood and tissue) could be explained by the tissue heterogeneity, suggesting that the pivotal genes might affect the immune infiltration in a tissue-specific manner. Of note, pivotal genes in both blood and tissue samples were associated with the proportion of CD8 T cells and resting memory CD4 T cells; such concordance suggests that pivotal genes might work in concert to suppress the recruitment of CD8 T cells (LY96, PIK3CD, ZBTB16 in tissue and MSH2 in blood) or promote the resting state of CD4 T cells (PIK3CD, HEBP2, ADAM8 in tissue and MSH2 in blood), thereby shaping the progression of IDD. Aside from macrophages (Silva et al. 2019), T cells (Wang and Samartzis 2014) and NK cells (Sato et al. 2002) that were proposed to participate in IDD, the potential roles of plasma cells in IDD are currently unclear, although plasma cells are crucial for maintaining the humoral immunity (D'Souza and Bhattacharya 2019). Based on the current results, the correlation between pivotal genes and plasma cells or NK cells might be specific characteristics for blood or tissue sample of IDD patients and might provide novel perspective on IDD diagnosis and treatment. Moreover, in our present study, myeloid cells of IDD patients also changed greatly. In addition, the genes LY96 (Tissières et al. 2009), ZBTB16 (Girard et al. 2013; Quaranta et al. 2006) and PIK3CD (Kok et al. 2009) which are related to myeloid cells were found to be key genes involved in IDD. These results suggested that the dysregulation of genes related to myeloid cells and their associated functions might indicate the pathogenesis of IDD. Indeed, we found that the “myeloid cell activation involved in immune response”, “myeloid leukocyte activation” and “myeloid leukocyte mediated immunity” processes were involved in the pathogenesis of IDD. These results are indicative of a probable implication of the myeloid cells in IDD pathogenesis. Up to date, the involvement of myeloid cells in the pathogenesis of IDD has not been systematically reported. A previous study on IDD indicated that microRNAs regulate apoptosis in myeloid cells; however, the regulated apoptosis pathways were not found to be IDD-specific (Yang 2022). The immune myeloid cells have been reported to regulate the extracellular matrix in cancer (Jiang and Lim 2016). Interestingly, previous studies have conveyed that the degeneration of interverbal disc is associated with excessive destruction of the outer disc extracellular matrix (ECM) due to the expression changes in matrix metalloproteinases (MMPs), which conducts to the decrease of intervertebral disc machinery and subsequent structural damage (Liu et al. 2020). Thus, we inferred that myeloid cells may be involved in IDD by releasing immune related immune and inflammatory markers and regulating the MMP proteins.

Our study may present some limitations. Indeed, contrary to the validation data GSE56081 which provided the pfirrmann disc grade for patients (Grades IV and V), the clinical grade data of the IDD patients from the GSE124272 are unavailable; since the dynamic changes of immune status in the context of IDD may change with the pathological stage, the results of our study may be taken with caution.

## Conclusions

The current study revealed a number of immune-associated pathways that might be responsible for the etiology of IDD, which might shed a light on in-depth researches on the pathogenesis of IDD in the context of immune response. The association between pivotal genes and immune cells are noteworthy and deserve further experimental validation.

## Supplementary Information

Below is the link to the electronic supplementary material.

Supplementary Figure S1. Overall design of the current study

Supplementary Table S1. GSEA–GO results (IDD patients versus HV)

Supplementary Table S2. GSEA–KEGG results (IDD patients versus HV)

## Data Availability

All data generated or analyzed during this study are included in this published article and its supplementary information files.

## Abbreviations

LBP: Low back pain
IDD: Intervertebral disc degeneration
IVD: Intervertebral disc
NP: Nucleus pulposus
NK: Natural killer
GSEA: Gene set enrichment analysis
WGCNA: Weighted correlation network analysis
BPNN: Back propagation neural network
